# Efficacy of an alternative positioning of intracardiac defibrillation catheters in atrial fibrillation ablation

**DOI:** 10.1002/joa3.70044

**Published:** 2025-03-21

**Authors:** Jumpei Ohashi, Tatsuya Hayashi, Shingo Yamamoto, Yusuke Ugata, Kenichi Sakakura, Hideo Fujita

**Affiliations:** ^1^ Division of Cardiovascular Medicine, Saitama Medical Center Jichi Medical University Saitama Japan

**Keywords:** atrial fibrillation, cryoballoon ablation, intracardiac defibrillation, intracardiac defibrillation catheter

## Abstract

**Background:**

In pulmonary vein isolation (PVI) for atrial fibrillation (AF), intraoperative defibrillation is often required. Intracardiac defibrillation catheters (ICDCs) are most effective when positioned to enclose the heart between the coronary sinus (CS) and right atrium (RA) (CS/RA configuration). However, achieving this positioning via the inferior vena cava (IVC) can be challenging, and alternative configurations remain underexplored.

**Methods:**

This study included patients with paroxysmal or persistent AF who underwent cryoballoon ablation followed by intracardiac cardioversion using an ICDC via the IVC. The catheter was initially positioned with distal electrodes in the CS and proximal electrodes in the IVC (CS‐only configuration). If cardioversion failed, the catheter was repositioned to place distal electrodes in the superior vena cava (SVC configuration). A maximum of 30 J of energy was used for all cardioversion attempts.

**Results:**

A total of 81 patients were included. Cardioversion in the CS‐only configuration restored sinus rhythm in 11% (9/81) of patients. Repositioning to the SVC configuration achieved successful cardioversion in 93.1% (67/72) of the remaining cases without complications. Patients requiring the SVC configuration had a significantly higher prevalence of persistent AF (33.3% vs. 80.6%; *p* = 0.045). No adverse events were observed following cardioversion in the SVC configuration.

**Conclusions:**

While the CS‐only configuration offers ease of placement, its efficacy is limited. Repositioning to the SVC configuration significantly enhances cardioversion success and represents a safer, more effective alternative for ICDC use during AF ablation.

## Graphical abstract

The SVC configuration, in which distal electrodes are placed in the SVC, demonstrated higher defibrillation success rates (93.1%). Furthermore, no adverse events were observed following cardioversion in this position.

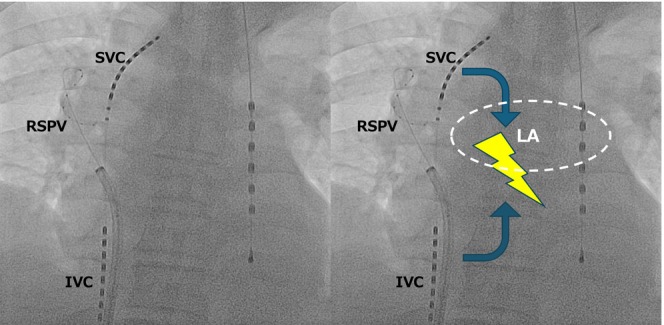

## 1 BACKGROUND

**FIGURE 1:**
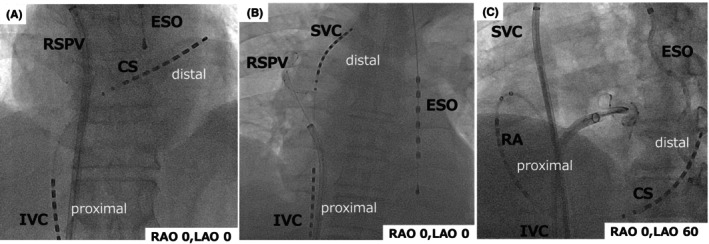
(A) The ICDC catheter in the CS‐only configuration is shown. The distal electrodes were positioned within the CS, while the proximal electrodes were located in the IVC. (B) The ICDC catheter in the SVC configuration is shown. The distal electrodes were positioned within the SVC, while the proximal electrodes were placed in the IVC. (C) The ICDC catheter in the RA/CS configuration is shown. The distal electrodes were positioned within the SVC, while the proximal electrodes were placed in the RA, forming an α‐loop configuration. CS, coronary sinus; ESO, esophagus; IVC, inferior vena cava; RA, right atrium; RSPV, right superior pulmonary vein.

Pulmonary vein (PV) isolation has become a standard procedure for the treatment of atrial fibrillation (AF).^1^, ^2^ During this procedure, cardioversion or defibrillation is often required to terminate atrial tachycardia (AT) or AF and restore sinus rhythm.^3^, ^4^, ^5^, ^6^ While external cardioversion and defibrillation are commonly used, they require the removal of surgical drapes, temporarily disrupting the sterile field and potentially increasing procedural time and complexity. Although the risk of skin burns is generally low, it is not negligible. Additionally, external cardioversion requires higher energy than internal cardioversion, which can lead to more significant patient movement. Excessive movement during the procedure can be problematic, particularly when catheter stability is crucial, and displacement of the 3D mapping system can also be a significant issue. To address these challenges, although there are some limitations related to access and regional availability, the intracardiac defibrillation catheter (ICDC) is often utilized due to its convenience and lower energy requirement for cardioversion, which minimizes patient movement and eliminates the risk of skin burns. Among the ICDC devices, one of the most widely used is the BeeAT (Japan Lifeline, Tokyo), along with the dedicated internal defibrillator (Shock AT, Japan Lifeline, Tokyo), which are commonly utilized in regions where they are available. This ICDC catheter is equipped with a total of 20 electrodes, including 16 wide‐band electrodes for defibrillation (8 distal and 8 proximal) and 4 additional intermediate electrodes, all capable of recording potentials. The recommended configuration for ICDC use involves placing the distal eight electrodes in the coronary sinus (CS) and the proximal eight electrodes in the right atrium (RA), forming an α‐loop position (CS/RA configuration) (Figure 1), which effectively lowers the defibrillation threshold.^7^

In recent years, considering safety, femoral vein access via the inferior vena cava (IVC) has become preferred over access through the subclavian or jugular veins via the superior vena cava (SVC). This is because femoral vein access eliminates the risk of potentially serious complications such as pneumothorax, hemothorax, and accidental puncture of the internal carotid and subcuticular artery, which can occur with the latter approaches.^8^, ^9^ However, achieving the CS/RA configuration using femoral vein access presents technical challenges and carries potential risks, such as cardiac tamponade due to CS injury or other causes if the catheter is manipulated excessively.^8^ In this context, there is still insufficient discussion and consensus on alternative catheter locations when CS/RA placement via IVC proves difficult.

## 2 METHODS

### 2.1 Study design

This study is a retrospective analysis of paroxysmal and persistent AF patients who underwent cryoballoon (CB) ablation at our institution (Saitama Medical Center, Jichi Medical University) between June 2020 and June 2023. Patients who did not undergo cardioversion or whose initial cardioversion was performed using internal cardioversion with standard CS/RA configuration or external cardioversion were excluded.

### 2.2 Internal cardioversion protocol

Defibrillation was performed by delivering current between the distal eight electrodes and proximal eight electrodes of the ICDC. Each electrode was 4 mm in length, with a 2 mm spacing between electrodes. A biphasic direct current was supplied between the distal and proximal electrodes, synchronized with the R‐wave of the surface electrocardiogram. The pulse duration ratio of the biphasic waveform was fixed at 6:4, and the pulse duration was automatically adjusted based on the impedance. The maximum energy output of the system was 30 J, which was exclusively used in this study. This method was chosen because the purpose of this study was not to compare CS/RA configuration but to evaluate the utility of different catheter positions. As a result, the defibrillation threshold (DFT) was not measured. In this study, internal cardioversion was initially performed in the CS‐only configuration, where only the distal eight electrodes of the ICDC were positioned within the CS. In this setup, the proximal electrodes were typically located in the IVC (Figure 1A). This configuration was chosen first because it simplifies catheter insertion and facilitates atrial pacing during pulmonary vein isolation.

If sinus rhythm was not restored after delivering a shock in the CS‐only configuration, an attempt was made at intracardiac defibrillation using the SVC configuration. In this setup, the distal electrode was positioned with reference to the bronchial bifurcation landmark, about 1 cm above the roof of the left atrium (LA), and slightly bent towards the LA side (Figure 1B). The proximal electrode was typically placed within the IVC. The intracardiac defibrillation from the SVC configuration was performed after a minimum 1‐min interval following the prior defibrillation attempt in the CS‐only configuration. If defibrillation failed in either configuration, external DC or catheter repositioning to the CS/RA position achieved sinus rhythm at the discretion of the operator.

### 2.3 Ablation procedure

Antiarrhythmic drugs were discontinued at least five half‐lives before the procedure. All patients underwent anticoagulation therapy with either warfarin or direct oral anticoagulants (DOACs) for at least 4 weeks before hospitalization. The procedure was performed under deep sedation using fentanyl and propofol. A multisensory esophageal temperature monitoring probe was employed in all cases. The ICDC was inserted into the CS via the IVC. Heparin was administered before transseptal puncture, and after catheter insertion into the LA, the activated clotting time (ACT) was maintained at >350 s. Transseptal puncture was performed using a radiofrequency needle (Scoper; Fukuda Denshi, Tokyo) and an 8.5‐Fr sheath (SL0; Abbott, Minneapolis). A steerable 15‐Fr sheath (FlexCath; Medtronic, Minneapolis) was advanced into the LA over a guidewire. A 28‐mm fourth‐generation cryoballoon (CB; Arctic Front Advance, Medtronic, Minneapolis) was introduced into the pulmonary veins (PVs) via the 15‐Fr steerable sheath, along with a 20‐mm spiral mapping catheter (Achieve; Medtronic, Minneapolis). The CB was inflated near the PV ostium and positioned to achieve complete occlusion at the PV entrance. After confirming complete occlusion with contrast injection, a cooling cycle lasting 180–240 s was applied. During CB application to the right PVs, phrenic nerve pacing was performed using a 7‐Fr circular mapping catheter (PV Mapping Catheter; Synaptic MEDICAL, Beijing) placed in the SVC via the SL0 sheath. CB application was terminated if phrenic nerve paralysis occurred or if temperatures fell below −60°C for the CB and 15°C for the esophagus. The endpoint for PV isolation was defined as the elimination of local PV potentials (entrance block) and the absence of local capture during pacing (exit block). If the time to isolation (TTI) exceeded 60 s or could not be visually identified, additional CB applications were performed at the operator's discretion. No additional radiofrequency ablation was conducted in this study.

### 2.4 Analysis of ECG

We also investigated the relationship between the success of defibrillation and P‐wave morphology on a 12‐lead electrocardiogram (ECG). P‐wave characteristics, including total P‐wave duration, initial P‐wave duration, terminal P‐wave duration, amplitude, and area in lead V1, were analyzed using a digital measurement tool (ECG1550; Nihon Kohden, Tokyo). ECG recordings were typically obtained during the first outpatient visit, approximately 1 month after the ablation procedure. Patients with recurrent atrial fibrillation or atrial tachycardia, as well as those with ongoing atrial pacing, were excluded from the analysis.

### 2.5 Statistical analysis

Data are presented as percentages for categorical variables and as mean ± standard deviation (SD) for continuous variables that follow a normal distribution. Categorical variables are presented as counts (percentages), and comparisons were made using the chi‐square test. The Shapiro–Wilk test was performed to assess the normality of continuous variables. For continuous variables that followed a normal distribution, comparisons were made using one‐way analysis of variance (ANOVA), and for those that did not follow a normal distribution, the Kruskal–Wallis test was used. A *p*‐value of <0.05 was considered statistically significant. All data were analyzed using SPSS for Windows (version 25, SPSS Inc., Chicago, Illinois).

## 3 RESULTS

**FIGURE 2:**
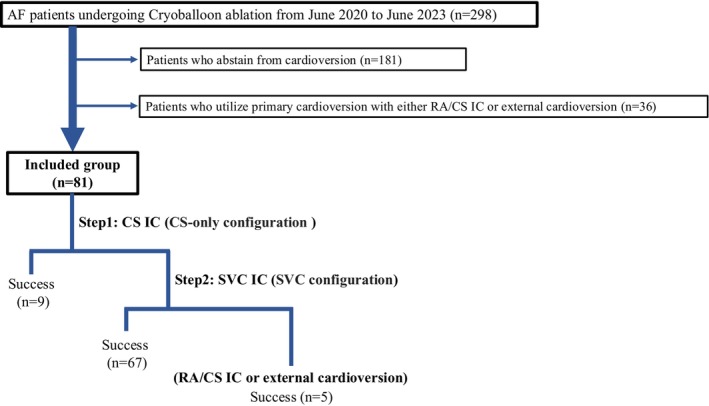
The flowchart of the study is presented. AF, atrial fibrillation; CS, coronary sinus; IC, internal cardioversion; SVC, superior vena cava; RA, right atrium.

Between June 2020 and June 2023, a total of 298 AF patients underwent CB ablation. Based on exclusion criteria, 217 patients were excluded, leaving 81 AF patients for the analysis (Figure 2). The mean age was 68 ± 12 years, with 49 male patients (64.5%). The mean left atrial diameter was 45 ± 7 mm, and 57 patients (75.0%) had persistent AF (Table 1). For patients in whom AF did not terminate after cryoablation, a 30‐J direct current (DC) shock was initially applied with the catheter positioned in the CS‐only configuration. Sinus rhythm was restored in 9 of 81 (11.0%) patients. In cases where CS‐only configuration cardioversion was unsuccessful (72 patients), the catheter was repositioned straight into the SVC configuration, and another 30‐J DC shock was administered. Sinus rhythm was successfully restored in 67 of 72 patients (93.1%). No adverse events were observed in any patient, including after DC shocks in the SVC configuration. For the remaining 5 patients who did not respond to SVC configuration DC shocks, external DC or catheter repositioning to the CS/RA position achieved sinus rhythm (Table 2).

**TABLE 1:** Comparison of patient clinical characteristics.

	All (*n* = 76)	CS‐only configuration successful group (*n* = 9)	SVC configuration successful group (*n* = 67)	*p*‐value
Age, year	68 ± 12	67 ± 13	69 ± 12	0.72
Male gender (%)	49 (64.5)	5 (55.6)	44 (65.7)	0.55
Body mass index (kg/m^2^)	23.9 ± 3.7	25.2 ± 4.4	23.7 ± 3.6	0.28
Hypertension (%)	54 (71.1)	7 (77.8)	47 (70.1)	0.64
Diabetes mellitus (%)	16 (21.1)	1 (11.1)	15 (22.4)	0.44
Dyslipidemia (%)	19 (25.0)	2 (22.2)	17 (25.4)	0.84
CHADS2 score	1.6 ± 1.2	1.4 ± 1.2	1.7 ± 1.2	0.62
Hb (g/dL)	14.3 ± 1.8	14.0 ± 1.5	14.3 ± 1.8	0.69
eGFR (mL/min/1.73m^2^)	63.4 ± 23.0	65.6 ± 15.1	63.1 ± 23.9	0.76
HD (%)	2 (4.5)	0 (0)	2 (3.0)	0.60
BNP (peg/mL)	213.4 ± 220.1	187.8 ± 140.4	216.8 ± 250.6	0.71
CRP (mg/dL)	0.24 ± 0.56	0.13 ± 0.11	0.25 ± 0.59	0.55
Persistent AF (%)	57 (75.0)	3 (33.3)	54 (80.6)	0.045
AF duration (month)	9.7 ± 11.8	4.4 ± 7.5	10.5 ± 12.1	0.15
LAD (mm)	44.5 ± 6.8	44.9 ± 7.0	44.5 ± 6.8	0.87
Antiarrhythmic drugs (%)	9 (11.8)	2 (22.2)	7 (10.4)	0.31
PMI (%)	2 (2.7)	0 (0)	2 (3.0)	0.62
V1 P‐wave positive amplitude (μV)	74.6 ± 39.0	95.5 ± 41.6	71.6 ± 38.0	0.095
V1 P‐wave negative amplitude (μV)	−37.7 ± 28.1	−43.3 ± 23.9	−36.8 ± 28.7	0.46
Total P‐wave duration (ms)	95.8 ± 20.8	86.8 ± 14.4	97.9 ± 21.3	0.17
P‐wave axis (°)	49.0 ± 31.9	44.1 ± 34.1	49.7 ± 31.8	0.62

*Note*: Data are expressed as the mean ± standard deviation or number (percentage).

Abbreviations: AF, atrial fibrillation; BNP, brain natriuretic hormone; CRP, C‐reactive protein; CS, coronary sinus; eGFR, estimated glomerular filtration rate; HD, hemodialysis; LAD, left atrial dimension; PMI, pacemaker implantation; RA, right atrium; SVC, superior vena cava.

**TABLE 2:** Success rate of cardioversion by IC protocol.

	All	Success group (%)	Failure group (%)
CS‐only configuration	81	9 (11.0)	72 (89.0)
SVC configuration	72	67 (93.1)	5 (6.9)

Abbreviations: CS, coronary sinus; IC, internal cardioversion; RA, right atrium; SVC, superior vena cava.

When comparing the group that achieved sinus rhythm in the CS‐only configuration (CS‐only success, *N* = 9) with the group requiring cardioversion in the SVC configuration (SVC success, *N* = 67), the SVC success group demonstrated a significantly higher prevalence of persistent AF compared with the CS‐only success group (3 [33.3%] vs. 54 [80.6%], *p* = 0.045) (Table 1). Echocardiographic comparisons are presented in Table 3.

**TABLE 3:** Comparison of echocardiogram findings.

	All (*n* = 76)	CS‐only configuration successful group (*n* = 9)	SVC configuration successful group (*n* = 67)	*p*‐value
LVEF (%)	56.8 ± 12.6	60.3 ± 12.1	56.4 ± 12.7	0.39
LVDd (mm)	46.0 ± 5.3	45.2 ± 5.6	46.1 ± 5.4	0.65
LVDs (mm)	30.6 ± 6.3	29.4 ± 5.2	30.8 ± 6.1	0.53
IVST (mm)	9.7 ± 1.5	9.7 ± 1.4	9.7 ± 1.5	0.91
LVPWT (mm)	9.6 ± 1.5	9.4 ± 1.1	9.6 ± 1.5	0.77
E/e'	12.3 ± 6.0	9.9 ± 3.1	12.6 ± 6.2	0.26
Moderate to severe MR (%)	8 (10.5)	0 (0)	8 (11.9)	0.27
Moderate to severe MS (%)	0	0	0	—
Moderate to severe AR (%)	0	0	0	—
Moderate to severe AS (%)	3 (3.9)	1 (11.1)	2 (3.0)	0.28

*Note*: Data are expressed as the mean ± standard deviation or number (percentage).

Abbreviations: AR, aortic valve regurgitation; AS, aortic valve stenosis; IVST, interventricular septal thickness; LVDd, left ventricular diameter at end diastole; LVDs, left ventricular diameter at end systole; LVEF, left ventricular ejection fraction; LVPWT, left ventricular posterior wall thickness; MR, mitral regurgitation; MS, mitral stenosis.

## 4 DISCUSSION

This study is the first to compare the defibrillation efficiency of an ICDC in various positions beyond the standard CS/RA configuration. The main findings are as follows:

- While the CS‐only configuration allows for easier catheter placement, its defibrillation efficiency is relatively low (11.0%).
- By contrast, the SVC configuration demonstrated higher defibrillation success rates (93.1%). Furthermore, no adverse events were observed following cardioversion in this position.
- Patients with successful conversion in the SVC configuration demonstrated a significantly higher prevalence of persistent AF compared to those with successful conversion in the CS‐only configuration.

These results highlight that the SVC configuration may serve as a reliable and safe alternative when the standard CS/RA configuration is not achievable, particularly in patients with challenging anatomical features.

### 4.1 Intracardiac defibrillation

During AF ablation, defibrillation is often essential and may be performed at various time points during and after the procedure. In some cases, multiple defibrillation attempts may be necessary. Therefore, it is important to evaluate methods of defibrillation carefully. While external DC cardioversion is a widely used approach, it requires higher energy levels, which can lead to more significant patient movement. Excessive movement during the procedure can be problematic, particularly when catheter stability is crucial, and any displacement of the 3D mapping system can also be a significant issue. Additionally, external cardioversion carries a small but non‐negligible risk of skin burns. Intracardiac cardioversion using an ICDC presents a valuable alternative. Intracardiac cardioversion for restoring sinus rhythm in AF has been performed for several decades.^10^, ^11^, ^12^ Although it is not yet a standardized method worldwide due to factors such as catheter availability, historical practice differences, and variations in healthcare infrastructure, intracardiac defibrillation offers significant advantages. It requires lower energy to terminate atrial arrhythmias compared with external DC, possibly minimizing patient movement, eliminating the risk of skin burns, and seamlessly integrating into electrophysiological studies (EPS) or ablation procedures. These benefits make it a practical and efficient option, improving procedural workflow and patient comfort.

However, the relationship between catheter placement ease and defibrillation efficacy remains unclear.^13^ The standard CS/RA configuration is considered the most efficient setup for ICDC placement when feasible, but achieving this configuration can sometimes be technically challenging, especially when patients have an enlarged atrium. In this study, we evaluated 81 AF patients undergoing CB ablation who required intracardiac cardioversion and found that the SVC configuration could serve as a viable alternative to the standard CS/RA configuration when the latter placement is not achievable.

### 4.2 ICDC position and efficacy

The ICDC delivers defibrillation energy by transmitting current between its distal and proximal electrodes.^14^ While the standard CS/RA position, where both atria are sandwiched between the electrodes, is theoretically considered the most effective configuration,^15^ previous studies have demonstrated that effective defibrillation can also be achieved with alternative placements. For instance, Plewan et al. reported that positioning the ICDC distal electrodes in the right pulmonary artery could successfully achieve defibrillation.^16^ Similarly, Alt et al. demonstrated that positioning the ICDC in the pulmonary artery was as effective as the CS/RA position due to the anatomical proximity of the pulmonary artery to the superior portion of the left atrium, allowing both atria to be placed between the electrodes.^15^

However, until this study, the efficacy of positioning the distal electrode in the SVC had not been thoroughly investigated. The results of this study suggest that during the IVC approach, the SVC configuration, considered the easiest to position compared with other configurations, is effective for defibrillation. When the distal electrode is positioned within the SVC, the anatomy indicates that only the right atrium lies between the distal and intermediate electrodes. Thus, the precise mechanism by which intracardiac defibrillation in the SVC position is effective remains unclear. One potential explanation is that the current generated by the distal electrode does not travel in a straight line directly to the proximal electrode. Instead, it follows a more circuitous path, potentially passing through the left atrium, which may enhance defibrillation efficiency.

Predictors of a high DFT in internal cardiac defibrillation include long‐standing AF, LA enlargement, and left ventricular enlargement.^17^, ^18^ The present study focused on patients undergoing CB ablation, with only a few having long‐standing AF. Consequently, no significant statistical correlation was observed between cardioversion failure and AF duration. Based on previous studies suggesting that RA‐LA conduction may be involved in defibrillation efficiency,^19^, ^20^, ^21^ we also analyzed P‐wave morphology during sinus rhythm to evaluate interatrial conduction characteristics in this study. However, no significant relationship was observed between P‐wave morphology and the defibrillation success rate (*p* = 0.095). This result may be attributed to the small sample size, the study's focus on success versus failure rather than the defibrillation threshold, and the inclusion of both paroxysmal and persistent atrial fibrillation in the study design. However, the study demonstrated that patients with successful cardioversion in the SVC configuration had a higher prevalence of persistent AF compared to those in the CS‐only configuration. Additionally, patients with successful cardioversion in the SVC configuration tended to have a longer AF duration compared to those with CS‐only configuration success (10.5 ± 12.1 vs. 4.4 ± 7.5 months, *p* = 0.154). These findings suggest that the SVC configuration may be preferable for patients with higher risk factors for elevated DFTs compared with the CS‐only configuration.

### 4.3 Clinical implications

The findings of this study hold significant clinical importance. The CS/RA configuration is highly effective for reducing the defibrillation threshold, as it allows for optimal placement of the catheter across both atria. However, catheter manipulation can be technically challenging, and excessive force may carry the risk of complications such as coronary sinus injury. In recent years, catheter‐based treatments in EPS/ABL have increasingly utilized lower limb venous access. This approach offers several advantages, including minimizing critical complications, reducing radiation exposure, and addressing cosmetic concerns associated with visible access points in the neck/clavicular region. However, this approach can make achieving the CS/RA position more difficult. Given these considerations, for patients with paroxysmal AF who may not initially require defibrillation, beginning the procedure with the CS‐only position is a reasonable and practical approach via IVC. This ensures that basic pacing remains achievable while simplifying catheter placement. However, our study demonstrated that defibrillation efficacy is relatively low in the CS‐only position.

Based on our findings, we propose the following strategy in AF ablation with ICDC via the IVC approach:

1. In cases where CS/RA positioning proves challenging, initiate ablation with the CS‐only configuration.
2. If AF persists during the procedure, reposition the catheter to the SVC configuration to perform an internal DC shock.

This approach avoids unnecessary manipulation to achieve the CS/RA configuration, enhancing both safety and efficiency during the procedure.

Nevertheless, the CS/RA position remains indispensable in specific cases. Its ability to observe both atrial potentials makes it particularly valuable for ablating non‐pulmonary vein (non‐PV) foci, such as those originating from the RA. Therefore, in complex cases where RA potentials are critical, the CS/RA configuration should remain the first‐choice configuration.

In summary, while the CS/RA position remains the gold standard for ICDC placement, our findings demonstrate that the SVC position is an effective and feasible alternative, particularly when catheter placement in the CS/RA position proves challenging.

ICDC is a valuable tool in electrophysiology; however, its availability may be limited in certain countries or regions, and cost‐related challenges could impact its use. While the current cost of ICDC may be higher compared with standard electrode catheters combined with external DC patches, wider recognition of its clinical utility could lead to a reduction in price over time. Therefore, we believe that every electrophysiologist should be well informed about the characteristics and advantages of ICDC.

### 4.4 Study limitations

This study was a single‐center, retrospective investigation with a relatively small sample size, limiting the generalizability of its findings. As a preliminary evaluation, the internal cardioversion protocol employed in this study used a fixed maximum energy output of 30 J for all cardioversions, and DFT measurements were not conducted. Consequently, direct comparisons with other techniques regarding efficacy could not be established. Cardioversion was initially performed using the CS‐only configuration, followed by DC shock in the SVC configuration. Although a minimum interval of 1 min was maintained between procedures to allow for a sufficient refractory period, the initial DC shock delivered in the CS‐only configuration may have influenced the outcomes of the subsequent shock delivered in the SVC configuration.

## 5 CONCLUSION

The novel approach of positioning the ICDC distal electrodes in the SVC was found to achieve rapid, safe, and effective cardioversion during pulmonary vein isolation in AF patients. This study highlights a new possibility for utilizing the ICDC in alternative positions beyond the standard CS/RA configuration.

## CONFLICT OF INTEREST STATEMENT

Authors declare no conflict of interest for this article.

## Data Availability

Available upon reasonable request.
